# Objectively measured light-intensity lifestyle activity and sedentary time are independently associated with metabolic syndrome: a cross-sectional study of Japanese adults

**DOI:** 10.1186/1479-5868-10-30

**Published:** 2013-03-04

**Authors:** Junghoon Kim, Kai Tanabe, Noriko Yokoyama, Hirofumi Zempo, Shinya Kuno

**Affiliations:** 1 Department of Sports Medicine, Graduate School of Comprehensive Human Sciences, University of Tsukuba, 305-8577, Tsukuba, Ibaraki, Japan; 2 Japan Society for the Promotion of Science, Tokyo, Japan

**Keywords:** Lifestyle activity, Light-intensity activity, Metabolic syndrome, Sedentary time

## Abstract

**Background:**

Reducing sedentary time and increasing lifestyle activities, including light-intensity activity, may be an option to help prevent metabolic syndrome (MetS). The purpose of the present study was to examine whether objectively measured light-intensity lifestyle activity and sedentary time is associated with MetS, independent of moderate–vigorous intensity physical activity (MVPA).

**Methods:**

The participants in this cross-sectional study were 483 middle-aged Japanese adults, aged 30–64 years. The participants were divided into those with or without MetS according to the Japanese criteria for MetS. A triaxial accelerometer was used to measure light-intensity lifestyle activity [1.6–2.9 metabolic equivalents (METs)] and sedentary time (≤1.5 METs). Logistic regression was used to predict MetS from the levels of light-intensity lifestyle activity and sedentary time with age, sex, smoking, calorie intake, accelerometer wear time, and MVPA as covariates.

**Results:**

The odds ratios (OR) for MetS in the highest and middle tertiles of light-intensity lifestyle activity were 0.44 [95% confidence interval (CI): 0.24 to 0.81] and 0.51 (95% CI: 0.29 to 0.89) relative to the lowest tertile, after adjustment for age, sex, smoking, calorie intake, accelerometer wear time and MVPA (*P*_trend_ = 0.012). Sedentary time was also associated with the risk of MetS (*P*_trend_ = 0.018). Among participants in the highest tertile of sedentary time, the risk of MetS was 2.27-times greater than that in the lowest tertile (95% CI: 1.25 to 4.11). The risk of MetS was not significantly increased in subjects in the middle tertile of sedentary time.

**Conclusions:**

We found that light-intensity lifestyle activity and sedentary time were significantly associated with the risk of MetS, independent of MVPA. The results of our study suggest that public health messages and guidelines should be refined to include increases in light-intensity lifestyle activity and/or decreases in sedentary time, alongside promoting MVPA, to prevent MetS.

## Introduction

Metabolic syndrome (MetS) is a cluster of risk factors that increase an individual’s risk of developing cardiovascular disease [1]. Individuals with MetS are at greater risk of developing type 2 diabetes [2,3] and atherosclerotic cardiovascular disease compared with individuals without MetS. The cardiovascular disease mortality rate is also higher among people with MetS [4,5].

Epidemiological studies have consistently shown that moderate–vigorous intensity physical activity (MVPA) is associated with the risk of MetS [6-9]. Most studies of physical activity have focused on MVPA in leisure time because it involves greater energy expenditure, increases physical fitness, and decreases the risk of MetS [9-12]. In Japan, The Exercise and Physical Activity Reference for Health Promotion report 2006 (EPAR2006) compiled the then current guidelines for health promotion and provides recommendations for physical activity for Japanese individuals [13]. These guidelines, aimed at preventing lifestyle-related diseases, recommend that adults accumulate 23 metabolic equivalent (MET)-h/week of MVPA exceeding 3 METs [13]. However, for most people it is difficult to meet the recommended amount of physical activity from only MVPA.

In fact, only ~30% of Japanese adults were reported to fulfill the recommended physical activity level [14], so other types of physical activity should be considered. It is also important to determine whether lifestyle activities, including light-intensity activities, can improve health [15-17]. Light-intensity activities, such as walking slowly around the home, store or office, and other housework or workplace activities [18,19], are the main determinant of variability in total daily energy expenditure [20,21].

Furthermore, it was reported that sedentary behavior is associated with MetS and its components, independent of MVPA, in studies in Western countries, including the USA and Australia [22-25]. Several studies that objectively measured sedentary time using accelerometers showed that these factors are associated with MetS risk factors in adults [15,26,27]. These observations suggest the need for more recommendations regarding the health benefits of increases in light-intensity lifestyle activity and decreases in sedentary time. Nevertheless, the potential influence of lifestyle activities and sedentary time on MetS is poorly understood, particularly in Japanese individuals. Thus, the purpose of the present study was to examine whether objectively measured light-intensity lifestyle activity and sedentary time is associated with MetS, independent of MVPA.

## Methods

### Participants

A description of the study participants, design and methods used in this study are reported in our previous study [28]. In brief, we performed a cross-sectional study of 521 healthy middle-aged Japanese adults without diabetes, cardiovascular disease or musculoskeletal diseases living in Tsukuba City who underwent medical examinations. Participants were recruited through advertisements placed in local newspapers. In this analysis, we excluded individuals with missing data on physical activity (n = 11), missing MetS components (n = 18) and missing dietary intake (n = 9). Therefore, 483 participants (179 men and 304 women) were included in this analysis [mean ± standard deviation (SD) age, 47.9 ± 9.0 years; body mass index (BMI), 25.6 ± 4.0 kg/m^2^]. The characteristics of the participants included in this analysis are presented in Table 1. This study was approved by the Ethical Committee of the Institute of Health and Sport Sciences and the Institute of Clinical Medicine at the University of Tsukuba. All participants provided written informed consent.

**Table 1:** Participant characteristics

**Demographics**	**Mean ± SD or %**
*N*	483
Sex (men, %)	37.1
Age (years)	47.9 ± 9.0
30-39 years (%)	22.2
40-49 years (%)	33.5
50+ years (%)	44.3
BMI (kg/m^2^)	25.6 ± 4.0
Obese (BMI ≥ 25 kg/m^2^, %)	51.3
***Physical activity parameters***	
Sedentary time (h/day)	4.6 ± 1.8
Light-intensity activity (METs-h/day)	12.8 ± 3.6
MVPA (METs-h/day)	3.6 ± 1.5
***Percentage of subject with***	
MetS	23.2
Abdominal obesity	52.3
Hypertension	50.1
Hyperglycemia	22.8
Dyslipidemia	21.5
Calorie intake (kcal/day)	1983.2 ± 310.6
Smoker (%)	11.6

*BMI*, body mass index; *MET*, metabolic equivalent; *MVPA*, moderate–vigorous intensity physical activity; *MetS*, metabolic syndrome; Abdominal obesity: waist circumference ≥ 85 cm in men, ≥ 90 cm in women; hypertension: systolic blood pressure ≥ 130 mmHg and/or diastolic blood pressure ≥ 85 mmHg; hyperglycemia: blood glucose ≥ 110 mg/dL; dyslipidemia: triglyceride ≥ 150 mg/dL and/or high-density lipoprotein cholesterol level < 40 mg/dL.

### Physical activity and sedentary time

Physical activity and sedentary time were measured using a triaxial accelerometer (HJA-350IT, Active style Pro, Omron Healthcare Co., Ltd.) [28,29]. To ensure the data were valid and reflected regular day-to-day activities, the participants were instructed to wear the accelerometer for 7 days while continuing regular daily activities [30]. The participants were instructed to wear the accelerometer on the right hip throughout the day from the time they woke up in the morning until they went to bed at night, except during showers or bathing.

All acceleration data were recorded as mean values of 1-min epochs. Periods of time in which accelerometer data were not recorded for ≥ 1 min were defined as the ‘non-wear’ time. Participants who did not record at least 600 min of activity every day for 7 days were excluded from further analyses [30]. Accelerometer data were calculated as the time spent in each of three different intensity levels for 7 days as follows: sedentary time, ≤ 1.5 METs; light-intensity lifestyle activity, 1.6–2.9 METs, and MVPA, ≥ 3 METs [31,32]. To examine the effects of different levels of light-intensity lifestyle activity on the risk of MetS, we divided participants into tertiles of light-intensity lifestyle activity (< 11.1, 11.2–14.5 and ≥ 14.6 MET-h/day) and sedentary time (< 3.5, 3.5–5.3 and ≥ 5.4 h/day). We confirmed the validity and reliability of the accelerometer in our previous study. The percentage of time that the accelerometer correctly discriminated sedentary (PC work), moderate (normal and brisk walking) and vigorous intensity (jogging) activity was > 95.5%, while the percentage for light-intensity activity (slow walking) was 81.8% [29].

### Anthropometric measurements

Body weight was measured to the nearest 0.1 kg using a digital scale without shoes. Height was measured to the nearest 0.1 cm using a wall-mounted stadiometer. BMI was calculated from body weight and height (kg/m^2^). Waist circumference (WC) was measured three times to the nearest 0.1 cm using a calibrated measuring tape at the midpoint of the lower costal margin; the mean value was used in the analyses.

### Metabolic syndrome components

Blood pressure was measured using an automated sphygmomanometer (SM-100; OMRON, Kyoto, Japan) with the participant in the seated position after resting for ≥ 10 min. A blood sample (~10 mL) was drawn from all participants after a ≥ 12-h fast.

The Japanese criteria for MetS were used to evaluate the prevalence of MetS [33,34], and consisted of abdominal obesity (WC ≥ 85 cm in men and ≥ 90 cm in women) as an essential criterion plus any two of the following three factors: (1) dyslipidemia [triglyceride (TG) ≥ 150 mg/dL and/or high density lipoprotein cholesterol (HDL-C) level < 40 mg/dL or specific treatment for these lipid abnormalities]; (2) blood pressure ≥ 130/85 mmHg or on drug treatment; and (3) fasting glucose ≥ 110 mg/dL. MetS components were also standardized by calculating Z scores for each participant [Z = (value – mean)/SD]. We constructed a continuous clustered MetS score from the sum of each Z score, similar to that done in a previous study [16], as follows: zWC + zBoold pressure [(systolic blood pressure + diastolic blood pressure)/2] + zFasting glucose + zTG + and inverted zHDL-C.

### Covariates

Energy intake was assessed using 3-day weighed dietary records. The food data of the dietary records were converted to energy and nutrient data by a dietician and were analyzed using Eiyoukun software version 4.0 (Kenpakusya, Tokyo, Japan). The participants also answered questions about smoking status, and the use antihypertensive and antidyslipidemic drugs using a general health questionnaire. Smoking status was classified as never or current smoking.

### Statistical analysis

Values are expressed as means ± standard deviation of the mean or percentage. Values of *P* < 0.05 were considered statistically significant. All data were analyzed using R version 2.14.0 [35]. Although we evaluated interactions by sex between light-intensity lifestyle activity or sedentary time, and the components of MetS, the interactions were not significant. Therefore, in analyses of physical activity and sedentary time, men and women were evaluated as a single group, as described in Additional file 1: Table S1 and Additional file 2: Table S2.

The statistical significance for linearity between light-intensity lifestyle activity and sedentary time categories was evaluated using Cochran-Armitage trend tests. Generalized linear models were used to predict the continuous components of MetS and clustered metabolic score based on light-intensity lifestyle activity and sedentary time. These models included age, sex, smoking, calorie intake and MVPA as covariates. Light-intensity activity and sedentary time were included in the analyses in 1-h units.

Logistic regression was also used to predict the prevalence of MetS and its components from the levels of light-intensity lifestyle activity and sedentary time. All logistic regression models included the following covariates: age, sex, smoking, calorie intake, accelerometer wear time and MVPA. We determined the variance inflation factors (VIF) for multicollinearity in all generalized linear models and logistic regression models as previously described [36], but these were not significant as all VIF values were < 10.

## Results

The characteristics of the participants are presented in Table 1. The present analysis included 483 Japanese population aged 30–64 years. Overall, 23.2% of the participants had MetS (Table 1). The mean sedentary time was 4.6 h/day. Light-intensity lifestyle activity and MVPA were 12.8 and 3.6 METs-h/day, respectively.

Table 2 shows the frequency of MetS and its components according to levels of light-intensity lifestyle activity. As shown in the table, the levels of light-intensity lifestyle activity were significantly and negatively associated with the frequencies of MetS (*P*_trend_ = 0.001), abdominal obesity (*P*_trend_ < 0.001) and dyslipidemia (*P*_trend_ < 0.001). By contrast, the frequencies of hypertension and hyperglycemia were not significantly different among levels of light-intensity lifestyle activity (Table 2). Table 2 also shows that sedentary time was significantly and positively associated with the frequencies of MetS (*P*_trend_ = 0.002), abdominal obesity (*P*_trend_ = 0.004) and dyslipidemia (*P*_trend_ < 0.001). On the other hand, the frequencies of hypertension and hyperglycemia were not significantly different among tertiles sedentary time (Table 2).

**Table 2:** Frequency (%) of MetS and its components according to tertiles of light-intensity lifestyle activity and sedentary time in daily life

	**Light-intensity lifestyle activity (METs-h/day)**			
	**<11.1 METs-h/day (n = 161)**	**11.2–14.5 METs-h/day (n = 161)**	**≥14.6 METs-h/day (n = 161)**	***P*-value for trend**
MetS (%)	34.8	19.3	15.5	0.001
Abdominal obesity (%)	67.7	46.3	43.1	<0.001
Hypertension (%)	51.6	50.3	48.3	0.641
Hyperglycemia (%)	26.9	19.3	22.4	0.449
Dyslipidemia (%)	33.5	20.5	10.6	< 0.001
	**Sedentary time (h/day)**			
	**< 3.5 h/day (n = 161)**	**3.5–5.3 h/day (n = 161)**	**≥ 5.4 h/day (n = 161)**	***P*-value for trend**
MetS (%)	15.5	20.3	34.4	0.002
Abdominal obesity (%)	41.6	54.1	61.9	0.004
Hypertension (%)	48.2	53.8	48.4	0.977
Hyperglycemia (%)	22.6	20.3	25.6	0.613
Dyslipidemia (%)	10.7	19.0	35.7	<0.001

Values are percentages. *P*-value for trend were calculated using the Cochran-Armitage Test. MetS, metabolic syndrome; *MET*, metabolic equivalent. Abdominal obesity: waist circumference ≥ 85 cm in men, ≥ 90 cm in women; hypertension: systolic blood pressure ≥ 130 mmHg and/or diastolic blood pressure ≥ 85 mmHg; hyperglycemia: blood glucose ≥ 110 mg/dL; dyslipidemia: triglyceride ≥ 150 mg/dL and/or high-density lipoprotein cholesterol level < 40 mg/dL.

Table 3 shows the results of the generalized linear models of light-intensity lifestyle activity and sedentary time. Light-intensity lifestyle activity was significantly associated with WC (β = −0.827; 95% CI: −1.518 to −0.137), HDL-C (β = 1.118; 95% CI: 0.188 to 2.049) and clustered MetS risk score (β = −0.249; 95% CI: −0.448 to −0.051), after adjusting for age, sex, smoking, calorie intake, accelerometer wear time and MVPA, but not with SBP, DBP, fasting glucose or TG. Sedentary time was significantly associated with WC (β = 1.034; 95% CI: 0.462 to 1.606), fasting glucose (β = 1.020; 95% CI: 0.015 to 2.025), TG (β = 5.815; 95% CI: 1.791 to 9.838), HDL-C (β = −1.491; 95% CI: −2.262 to −0.720) and zMetS (β = 0.329; 95% CI: 0.164 to 0.494), but not with SBP or DBP.

**Table 3:** Multivariable associations between light-intensity lifestyle activity or sedentary time and MetS components

	**β-coefficients (95% CI)**			
	***Light-intensity activity (h/day)***	***P*-value**	***Sedentary time (h/day)***	***P*-value**
WC (cm)	−0.827 (−1.518 to −0.137)	0.041	1.034 (0.462 to 1.606)	0.003
SBP (mmHg)	−3.035 (−6.695 to 0.625)	0.209	2.105 (−0.957 to 5.168)	0.177
DBP (mmHg)	−0.215 (−0.956 to 0.525)	0.576	0.264 (−0.355 to 0.883)	0.414
Fasting glucose (mg/dL)	−0.790 (−1.993 to 0.413)	0.097	1.020 (0.015 to 2.025)	0.047
TG (mg/dL)	−3.582 (−8.424 to 1.259)	0.107	5.815 (1.791 to 9.838)	0.017
HDL-C (mg/dL)	1.118 (0.188 to 2.049)	0.020	−1.491 (−2.262 to −0.720)	0.001
zMetS	−0.249 (−0.448 to −0.051)	0.006	0.329 (0.164 to 0.494)	0.001

Adjusted for age, sex, smoking status, calorie intake, accelerometer wear time and moderate–vigorous intensity physical activity. *MetS*, metabolic syndrome; *WC*, waist circumference; *SBP*, systolic blood pressure; *DBP*, diastolic blood pressure; *TG*, triglyceride; *HDL-C*, high-density lipoprotein cholesterol.

The results of logistic regression analyses comparing the prevalence of MetS across tertiles of light-intensity lifestyle activity and sedentary time are shown in Tables 4 and 5, respectively. The odds ratios (OR) for MetS in the highest and middle tertiles of light-intensity lifestyle activity were 0.44 (95% CI: 0.24 to 0.81) and 0.51 (95% CI: 0.29 to 1.89) relative to the lowest tertile (Table 3, *P*_trend_ = 0.012). The levels of light-intensity lifestyle activity were also significantly associated with a reduced risk for abdominal obesity (*P*_trend_ = 0.005) and dyslipidemia (*P*_trend_ = 0.016).

**Table 4:** Associations between light-intensity activity and the prevalence of MetS and its components

	***Light-intensity activity category***			
**Outcomes**	**<11.1 METs-h/day (n = 161)**	**11.2–14.5 METs-h/day (n = 161)**	**≥14.6 METs-h/day (n = 161)**	***P*-value for trend**
**MetS**	1 (Reference)	0.51 (0.29 to 0.89)*	0.44 (0.24 to 0.81)*	0.012
**Abdominal obesity**	1 (Reference)	0.46 (0.28 to 0.76)*	0.50 (0.30 to 0.84)*	0.005
**Hypertension**	1 (Reference)	0.98 (0.61 to 1.58)	0.97 (0.59 to 1.60)	0.993
**Hyperglycemia**	1 (Reference)	0.68 (0.38 to 1.23)	0.94 (0.51 to 1.72)	0.394
**Dyslipidemia**	1 (Reference)	0.68 (0.39 to 1.17)	0.39 (0.20 to 0.74)*	0.016

*P < 0.05; adjusted for age, sex, smoking status, calorie intake, accelerometer wear time and moderate–vigorous intensity physical activity. *OR*, odds ratio; *CI*, confidence interval; *MetS*, metabolic syndrome; *MET*, metabolic equivalent. Abdominal obesity: waist circumference ≥ 85 cm in men and ≥ 90 cm in women; hypertension: systolic blood pressure ≥ 130 mmHg and/or diastolic blood pressure ≥ 85 mmHg; hyperglycemia: blood glucose ≥ 110 mg/dL; dyslipidemia: triglyceride ≥ 150 mg/dL and/or high-density lipoprotein cholesterol level < 40 mg/dL.

**Table 5:** Associations between sedentary time and the prevalence of MetS and its components

	***Sedentary time category***			
**Outcomes**	**< 3.5 h/day**	**3.5–5.3 h/day**	**≥ 5.4 h/day**	***P*-value for trend**
	**(n = 161)**	**(n = 161)**	**(n = 161)**	
**MetS**	1 (Reference)	1.30 (0.71 to 2.38)	2.27 (1.25 to 4.11)*	0.018
**Abdominal obesity**	1 (Reference)	1.47 (0.92 to 2.36)	1.72 (1.03 to 2.86)*	0.091
**Hypertension**	1 (Reference)	1.20 (0.76 to 1.91)	0.93 (0.57 to 1.53)	0.540
**Hyperglycemia**	1 (Reference)	0.82 (0.46 to 1.46)	1.10 (0.61 to 1.99)	0.607
**Dyslipidemia**	1 (Reference)	1.68 (0.88 to 3.24)	3.00 (1.60 to 5.64)*	0.002

*P < 0.05; adjusted for age, sex, smoking status, calorie intake, accelerometer wear time and moderate–vigorous intensity physical activity. *OR*, odds ratio; *CI*, confidence interval; *MetS*, metabolic syndrome; *MET*, metabolic equivalent. Abdominal obesity: waist circumference ≥ 85 cm in men, ≥ 90 cm in women; hypertension: systolic blood pressure ≥ 130 mmHg and/or diastolic blood pressure ≥ 85 mmHg; hyperglycemia: blood glucose ≥ 110 mg/dL; dyslipidemia: triglyceride ≥ 150 mg/dL and/or high-density lipoprotein cholesterol level < 40 mg/dL.

We also found higher risks for MetS, abdominal obesity and dyslipidemia in subjects in the highest tertile of sedentary time after adjusting for age, smoking, calorie intake, accelerometer wear time and MVPA. This association was significant for subjects in the highest tertile of sedentary time compared with the lowest tertile, but not in the middle tertile (Table 5).

## Discussion

In the present study, we determined the effects of objectively measured light-intensity lifestyle activity and sedentary time on the prevalence of MetS in Japanese adults. The major finding of our study is that both light-intensity lifestyle activity and sedentary time are significantly associated with MetS, independent of MVPA. Most of the earlier studies investigating the relationship between physical activity and MetS focused on self-reported MVPA in leisure time [6-9]. To prevent lifestyle-related diseases, the EPAR2006 recommends that adults engage in 23 METs-h/week of MVPA [13]. However, for most people it is difficult to achieve this level of MVPA in leisure time. In 2009, Shibata et al. reported that only ~30% of Japanese adults engaged in the recommended level of MVPA [14]. On the other hand, several recent studies have revealed that lifestyle activity in daily life is associated with health benefits in adults [15-17]. For example, Camhi et al. [17] reported that the accumulation of light-intensity lifestyle activity measured using an accelerometer is independently associated with lower odds for certain cardiometabolic risk factors. Therefore, the results of this study and previous studies suggest that public health messages and guidelines should be updated to promote light-intensity lifestyle activity and decrease sedentary time, alongside increasing MVPA.

Our results are consistent with those of another study in which higher light-intensity activity was associated with decreased 2-h plasma glucose in Australian adults without diagnosed diabetes [15]. Another study has noted that light-intensity lifestyle activity measured using an accelerometer is associated with cardiometabolic risk factors [16]. The results of the present and other studies suggest that accumulating light-intensity lifestyle activity could be a convenient approach to increase physical activity for the prevention of MetS. For example, increasing lifestyle activities, including light-intensity activities (e.g., slowly walking around the home, store or office) [18,19], may be an option to increase overall physical activity to help prevent the MetS. Although lifestyle activities may not be vigorous in terms of aerobic effort, they do account for a considerable proportion of the total daily activity, and represent important sources of energy expenditure [20,21]. These activities may also contribute to the amount of daily activity required to improve health. Although MVPA is an important component of the public health message for preventing MetS, interventions aimed at replacing sedentary behavior with light-intensity activities may be more successful, particularly since ~70% of Japanese people do not conduct adequate amounts of physical activity to realize any health benefits [14]. However, it is important to consider that a greater amount of light-intensity lifestyle activity may be required to prevent MetS, as compared with MVPA [12].

In this study, we also determined the impact of sedentary time on the prevalence of MetS. The prevalence of MetS was approximately 2.27-times higher among in the highest tertile of sedentary time versus the lowest tertile. These results are consistent with those of earlier studies that examined the associations among sedentary time, obesity, MetS and mortality [22-25,37]. Several factors could explain this association between sedentary time and MetS. First, sedentary time may independently predict adverse health conditions. Ford et al. [22] and Bertrais et al. [25] also reported significant associations of television viewing time and duration of computer use with MetS, independent of MVPA. Furthermore, several studies have shown that objectively measured sedentary time is associated with MetS risk factors [26,27]. Thus, dual approaches may be necessary to increase someone’s physical activity or reduce sedentary time. Second, the impact of sedentary time on the risk of MetS may be the reverse of that of light-intensity activity. However, a common trait of these studies, as described above, did not estimate light-intensity activity, which may confound the association between sedentary time and health outcome. Consistent with previous studies, we found only weak significant association between sedentary time and MVPA (*R*^2^ = 0.244), but sedentary time was more strongly associated with light-intensity lifestyle activity (*R*^2^ = 0.78). The proportion of participants in the highest tertile of lifestyle activity increased from 2.5% to 15.2%, and 79.2% showed a change in category from the highest to middle or lowest tertiles of sedentary time (*P* < 0.05, data not shown). Moreover, when we statistically controlled for differences in objectively measured light-intensity lifestyle activity, the association between sedentary time and MetS lost its significance in the logistic regression model (*P* > 0.1, data not shown). Accordingly, the results of our study suggest that the effects of sedentary time on the risk of MetS are at least partly due to differences in light-intensity lifestyle activity.

### Strengths and limitations

The important strength of our study is that physical activity and sedentary time were objectively measured using triaxial accelerometers. However, accelerometers are not sensitive to all activities such as biking, standing and upper-body movement. The Sedentary Behaviour Research Network (SBRN) defines sedentary behavior as any waking behavior characterized by an energy expenditure of ≤ 1.5 METs while in a sitting or reclining posture [32]. However, in this study, we defined as sedentary time and light-intensity activity based on intensity levels (METs) as mean values of 1-min epochs. Therefore, it is possible we misclassified some instances of light-intensity activity, sedentary time and non-wear time.

A major limitation of this study is that we cannot infer causality or specify the direction of the effect because of its cross-sectional design. The prevalence of MetS in our subjects (men: 34.6%; women: 16.4%) was higher than that in a general population of Japanese individuals aged 30–69 years (men: 26.3%; women: 8.3%) included in the National Health and Nutrition Examination Survey (2009) [38]. Furthermore, the proportion of individuals who achieved the recommended MVPA level was higher in our study (52.2%) than in the general Japanese population (30.1%) [38]. Therefore, our current findings may not be generalizable to the Japanese population. In this study, the mean sedentary time was approximately 2–3 h/day lower in our participants than that reported in study performed in the USA and Australia [22,27,39]. This may also represent a limitation of our study, although this may also be due to differences in accelerometer wear time, cut-off values for sedentary activity, and the methods used to enrol the participants.

Although we controlled for several covariates, there may be residual confounding by other variables, such as sociocultural factors, family history of metabolic diseases, alcohol intake and physical fitness, which may partly explain our findings. Nevertheless, we took into account factors directly associated with the prevalence of MetS including MVPA, calorie intake and smoking [40,41]. Despite these limitations, our findings are important from a public health perspective, particularly in terms of designing interventions aimed at promoting physical activity by focusing on lifestyle activity among middle-aged Japanese individuals.

## Conclusion

In conclusion, we found that light-intensity lifestyle activity and sedentary time were associated with the risk of MetS, independent of levels of MVPA. Public health messages and guidelines should be refined to include increases in light-intensity lifestyle activity and/or decreases in sedentary time, in addition to promoting MVPA. The effectiveness of promoting light-intensity lifestyle activity on the prevention and treatment of MetS needs to be confirmed in longitudinal and intervention studies.

## Abbreviations

BMI: Body mass index; CI: Confidence interval; DBP: Diastolic blood pressure; EPAR2006: Exercise and Physical Activity Reference for Health Promotion report 2006; HDL-C: High-density lipoprotein cholesterol; MetS: Metabolic syndrome; MET: Metabolic equivalent; MVPA: Moderate–vigorous intensity physical activity; OR: Odds ratio; SBP: Systolic blood pressure; TG: Triglyceride; VIF: Variance inflation factor; WC: Waist circumference

## Competing interests

The authors declare that they have no competing interests.

## Authors’ contributions

JK designed the study, developed the research plans and instruments, conducted focus groups and helped write the manuscript. KT and NY transformed the accelerometer data and provided advice on statistical analyses. HZ helped to measure physical activity. SK was jointly responsible for the concept and design of the study. All authors read and approved the final version of the manuscript for publication.

## Supplementary Material

Additional file 1: Table S1 Interactions by sex between light-intensity lifestyle activity and the components of MetS. Click here for file

Additional file 2: Table S2 Interactions by sex between sedentary time and the components of MetS. Click here for file
