# Supplementary material for: Objectively measured light-intensity lifestyle activity and sedentary time are independently associated with metabolic syndrome: a cross-sectional study of Japanese adults
Source: Int J Behav Nutr Phys Act. 2013 Mar 4;10:30. doi: 10.1186/1479-5868-10-30 (PMC3599104; doi:10.1186/1479-5868-10-30)
Supplement: Additional file 1: Table S1 — Interactions by sex between light-intensity lifestyle activity and the components of MetS. [file 1479-5868-10-30-S1.docx]

| Table S1. Interactions by sex between light-intensity lifestyle activity and the components of MetS | | | |
| --- | --- | --- | --- |
|  | **β-coefficients (95% CI)** | |  |
|  | Light-intensity lifestyle activity | Interaction effect (light-intensity lifestyle activity × sex) | *Adjusted R*² |
| Waist circumference (cm) | −0.867 (−1.562 to −0.172)* | 0.672 (−0.714 to 2.057) | 0.076 |
| SBP (mmHg) | −2.795 (−6.48 to 0.889) | −4.069 (−11.403 to 3.264) | 0.038 |
| DBP (mmHg) | −0.255 (−1.001 to 0.491) | 0.677 (−0.808 to 2.162) | 0.092 |
| Fasting glucose (mg/dL) | −0.848 (−2.058 to 0.363) | 1.076 (−1.341 to 3.493) | 0.128 |
| Triglyceride (mg/dL) | −3.474 (−8.354 to 1.405) | −1.829 (−11.541 to 7.883) | 0.097 |
| HDL-C | 1.106 (0.168 to 2.044)* | 0.21 (−1.656 to 2.077) | 0.089 |
| zMetS | −0.258 (−0.458 to −0.058)* | 0.148 (−0.25 to 0.547) | 0.162 |
| Adjusted for age, sex, smoking, calorie intake, accelerometer wear time, and MVPA. | | | |
