# Supplementary material for: Objectively measured light-intensity lifestyle activity and sedentary time are independently associated with metabolic syndrome: a cross-sectional study of Japanese adults
Source: Int J Behav Nutr Phys Act. 2013 Mar 4;10:30. doi: 10.1186/1479-5868-10-30 (PMC3599104; doi:10.1186/1479-5868-10-30)
Supplement: Additional file 2: Table S2 — Interactions by sex between sedentary time and the components of MetS. [file 1479-5868-10-30-S2.docx]

| Table S2. Interactions by sex between sedentary time and the components of MetS | | | |
| --- | --- | --- | --- |
|  | **β-coefficients (95% CI)** | |  |
|  | Sedentary time | Interaction effect (sedentary time × sex) | *Adjusted*  *R*² |
| Waist circumference (cm) | 1.081 (0.498 to 1.664)* | −0.481 (−1.602 to 0.639) | 0.089 |
| SBP (mmHg) | 1.917 (−1.205 to 5.038) | 1.902 (−4.087 to 7.89) | 0.035 |
| DBP (mmHg) | 0.291 (−0.34 to 0.922) | −0.266 (−1.477 to 0.944) | 0.092 |
| Fasting glucose (mg/dL) | 1.134 (0.113 to 2.154)* | −1.234 (−3.202 to 0.733) | 0.133 |
| Triglyceride (mg/dL) | 6.065 (1.964 to 10.165)* | −2.522 (−10.389 to 5.345) | 0.109 |
| HDL-C | −1.528 (−2.314 to −0.743)* | 0.377 (−1.131 to 1.884) | 0.106 |
| zMetS | 0.35 (0.182 to 0.517)* | −0.207 (−0.529 to 0.114) | 0.180 |
| Adjusted for age, sex, calorie intake, smoking, accelerometer wear time, and MVPA. | | | |
